# Evaluation of the Antimicrobial Activity of ZnO Nanoparticles against Enterotoxigenic *Staphylococcus aureus*

**DOI:** 10.3390/life12101662

**Published:** 2022-10-20

**Authors:** Reham M. El-Masry, Dalia Talat, Shahira A. Hassoubah, Nidal M. Zabermawi, Nesreen Z. Eleiwa, Rasha M. Sherif, Mohammed A. S. Abourehab, Randa M. Abdel-Sattar, Mohammed Gamal, Madiha S. Ibrahim, Ahmed Elbestawy

**Affiliations:** 1 Directorate of Veterinary Medicine, El-Gharbia 31515, Egypt; 2 Department of Microbiology, Faculty of Veterinary Medicine, Damanhour University, Damanhour 22511, Egypt; 3 Department of Biological Sciences, Microbiology, King Abdulaziz University, Jeddah 21589, Saudi Arabia; 4 Animal Health Research Institute, Agriculture Research Center (ARC), Giza 12618, Egypt; 5 Laboratory of Directorate of Veterinary Medicine, Kafr El-Sheikh 33516, Egypt; 6 Department of Pharmaceutics, College of Pharmacy, Umm Al-Qura University, Makkah 21955, Saudi Arabia; 7 Department of Pharmaceutics and Industrial Pharmacy, College of Pharmacy, Minia University, Minia 61519, Egypt; 8 Biomedical Sciences Department, College of Pharmacy, Shaqra University, Shaqra 11961, Saudi Arabia; 9 Pharmaceutical Analytical Chemistry Department, Faculty of Pharmacy, Beni-Suef University, Alshaheed Shehata Ahmad Hegazy St., Beni-Suef 62514, Egypt; 10 Department of Poultry and Fish Diseases, Faculty of Veterinary Medicine, Damanhour University, Damanhour 22511, Egypt

**Keywords:** Gram-positive bacteria, nanoparticles, enterotoxin A, MIC

## Abstract

*Staphylococcus aureus* (*S. aureus*) is a Gram-positive bacteria considered one of the leading causes of community and hospital-acquired illnesses or public health concerns. Antibiotic resistance in this microorganism is one of the greatest issues in global health care. The use of metal nanoparticles and their oxides is one of the potential approaches to combating bacteria resistance to antibiotics. The antibacterial properties of ZnO NPs against enterotoxigenic *S. aureus* were studied. ZnO NPs were tested in vitro by agar diffusion test. They resulted in 26 and 22 mm zones of inhibition for a size of 20 nm and a concentration of 20 mM against 10^5^ and 10^7^ CFU/mL *S. aureus*, respectively. The MIC of ZnO NPs of various sizes, 20 and 50 nm, with 10^5^ CFU/mL was 2.5 and 5 mM, respectively. MIC with 10^7^ CFU/mL was five mM for 20 and 50 nm ZnO NPs. Further, the highest growth reduction percentage, 98.99% in the counts of *S. aureus* was achieved by ZnO NPs of size 20 nm and concentration of 10 mM. Moreover, the obtained ELISA results indicated a significantly decreased concentration of enterotoxin A with all concentrations and sizes of ZnO NPs. PCR analysis showed a significant effect on *sea* gene in response to ZnO NPs treatments leading to loss of the gene, unlike the unaffected nuc gene. Moreover, morphological changes and cell shape distortion were detected by scanning electron microscope for bacterial cells treated with ZnO NPs.

## 1. Introduction

Staphylococci are significant opportunistic infections in both human and animal medicine and are part of the typical flora of mammals and birds’ skin and mucous membranes [1]. *Staphylococcus aureus* is a potent Gram-positive bacterium. It has long been considered a public health concern [2]. *S. aureus* is a cause of infectious illness and produces a category of highly toxic proteins known as Staphylococcal enterotoxins [3]. Staphylococcal enterotoxin A (SEA) is responsible for most food poisoning caused by *S. aureus*, posing a concern to human health and resulting in various foodborne disorders [4].

*S. aureus*, like other bacteria, has a remarkable ability to resist any antibiotic to which it has been exposed [5]. Metal-based nanoparticles are commonly used in biomedical sciences and engineering [6,7]. These particles were used as an alternative to antibiotics to conquer microbial resistance because of their antibacterial effectiveness against Gram-positive and negative bacteria [8,9,10,11]. Nanoparticles use methods of action that differ from traditional treatments to target a variety of biomolecules that impede strain resistance development. [12,13].

ZnO NPs were investigated as an alternative antibiotic to improve antibacterial action on pathogenic strains. They have unique physicochemical characteristics that could affect microorganisms’ biological and toxicological responses. The main mechanisms underlying their antibacterial action are metal ion release, particle adsorption, and the creation of reactive oxygen species [14,15]. Various methods have been used to measure and investigate antimicrobial action in vitro: disk diffusion, broth dilution and the microtiter plate-based technique [16,17]. Various PCR-based molecular analytical tools such as end-point PCR, RT PCR, multiplex PCR and isothermal amplification of particular target DNA series were effectively used to quickly identify *S. aureus* [18]. Here, the antibacterial action of ZnO NPs on enterotoxigenic *S. aureus* was studied to determine effects on the bacterial growth rate, enterotoxin A production and *sea* gene.

## 2. Materials and Methods

Ethical Approval: All investigations and methods were adapted to the rules and endorsed by the Local Ethics Committee of the Animal Health and Welfare of Damanhour University. The Ethical Approval Code was DMU/VetMed-2021-/0158.

### 2.1. Bacterial Strain

*S. aureus* isolate was purchased from Animal Health Research Institute (Dokki, Giza). It was identified biochemically [19,20] and serologically by latex agglutination test Dry Spot kit (Staphytect plus, ThermoFisher Scientific, Hampshire, England) (Oxoid, 1990). Specific primers were used for the characterization of *S. aureus* by recognition of the *nuc* gene as illustrated in Table 1. DNA extraction and amplification reaction were carried out based on Valihrach et al. [21] and Cho et al. [22].

The bacterial strain was maintained in tryptic soy agar (Merck, Taufkirchen, Germany). Four to five separated colonies of the examined strain were collected via a sterilized inoculation loop and put into tubes of sterile peptone water 0.1% (Merck, Taufkirchen, Germany) (5 mL in each) and later incubated at 37 °C/24 h [24]. To measure the cell concentration, dilutions to 10^10^ of this culture were put in Baired Parker agar (Merck, Taufkirchen, Germany). The cell count for *S. aureus* was modified to 10^5^ CFU/mL and 10^7^ CFU/mL using the tube dilution method [25]. The number of CFU/mL was considered the infective dose to be inoculated into the broth. It is assumed that a minimal concentration of *S. aureus* of 10^5^ CFU/mL is needed for SEs production [26,27].

### 2.2. Preparation of ZnO NPs

ZnO NPs with a size of 20 and 50 nm were obtained from Nano Tech., Egypt, for Photo-Electronics based on NT-ZONP brand with a certificate of analysis. The physical properties of ZnO NPs were white; powder; spherical shape (TEM); stable colloid in a mixture of methanol, chloroform and water; optical properties (Abs.) of λ_max_ = 301 nm and 380 nm and average size (TEM) of 20 ± 5–50 ± 5 nm. Different concentrations were prepared, including concentrations of 2.5, 5, 10 and 20 mM. ZnO powder and nanoparticles were first sterilized at 160 °C for three hours, then distributed in distilled water (Milli-Q^®^, Millipore Corporation, Bedford, MA, USA), to prevent particle aggregation and deposition. The sample is aggressively vortexed for 10 minutes, followed by 30 minutes of sonication. The obtained suspensions (100 mL with a 1 M concentration) were regarded as a stock solution to be diluted and utilized for bacterial susceptibility testing [28].

### 2.3. Determination of the Effects of ZnO NPs against nuc Gene by PCR

Specific primers were used for the characterization of *S. aureus* by detection of the *nuc* gene for treated *S. aureus* with different sizes and concentrations of ZnO NPs as demonstrated in Table 1. DNA extraction was carried out based on Valihrach et al. [21]. A volume of 1 ml of overnight tryptic soy broth (TSB) culture or one loopful of *S. aureus* strain cultivated on tryptic soy agar (37 °C, stationary cultivation) and rinsed with 1 ml of sterile physiological solution was centrifuged at 14,000× *g* for 10 minutes at 4 °C. After the supernatant was removed, the pellet was resuspended in 0.2 ml of sterile distilled water. The sample was incubated at 100 °C for 20 min before being centrifuged at 17,000× *g* at 4 °C for 6 min to produce a supernatant that could be utilized immediately in a PCR reaction or frozen at −20 °C for future use. The amplification reaction was performed according to Cho et al. [22] using 25 μL of PCR mixture containing 3 μL of boiled cell lysate, 200 M of desoxynucleotide triphosphate (dNTP mixture), 1.4 U of Taq DNA polymerase (Biotools, Madrid, Spain), buffer (20 mM Tris-HCl pH 8.4, 50 mM KCl and 3 mM MgCl2, Biotools) and 20 M of each primer (*nuc*). Furthermore, the PCR condition was as follows: denaturation at 94 °C for 5 min, followed by 25 cycles of denaturation at 94 °C for 45 sec, annealing at 55 °C for 45 sec, and a final extension at 72 °C for 10 min. PCR amplified products were examined using 1.5% agarose gel electrophoresis stained with ethidium bromide and viewed and captured using a UV transilluminator. The fragment sizes were determined using a 100 bp plus DNA Ladder (Qiagen, Hilden, Germany).

### 2.4. Antimicrobial Activity of ZnO NPs

Agar well diffusion assay was applied to test the antimicrobial action on ZnO NPs of various sizes (20 and 50 nm) and concentrations (2.5, 5, 10 and 20 mM) against *S. aureus* (10^5^, 10^7^ CFU/mL). *S. aureus* was cultured in nutrient broth at 37 °C. The bacterial inoculums of 100 μl either 10^5^ or 10^7^ CFU/mL were streaked on the nutrient agar, and 100 μL of the tested ZnO NPs was poured into a central well in the plates. The plates were preserved at 37 °C for 24 h. The inhibitory zone was determined around the well (mm) [29].

### 2.5. Minimum Inhibitory Concentrations (MIC) of ZnO NPs

The modified microdilution method was used to determine the MIC of ZnO NPs suspension against *S. aureus*. A volume of 20 µL of 24-hour-old bacterial culture 10^5^ and 10^7^ CFU/mL of *S. aureus* was put in a 96-well plate and then 100µL of ZnO NPs suspensions of various sizes (20 and 50 nm) and concentrations (2.5, 5, 10 and 20 mM) were put on it in addition to an equivalent amount of Mueller–Hinton broth. As a control, a particle-free solution was employed. The plates were incubated overnight. A volume of 20 µL of p-iodonitro-tetrazolium violet aqueous solution (INT, Sigma-Aldrich), with a concentration of 4% w/v, was put into each well as a marker of bacteria. MIC was described as the smallest ZnO NPs concentration that prevented microbial growth [30].

### 2.6. Growth Inhibitory Effects of ZnO NPs against S. aureus

One hundred µL from each prepared nutrient broth inoculated with *S. aureus* (10^5^ and 10^7^ CFU/mL) against ZnO NPs at variable sizes (20 and 50 nm) and concentrations (5 and 10 mM) were spread on Baired Parker agar (Merck, Germany). The plates were preserved at 37 °C for 48 h. Black, glossy colonies were counted and expressed as colony forming units (log CFU/g) with tight white edges and a clear halo zone extending into the opaque medium [31].

### 2.7. Determination of Effects of ZnO NPs against S. aureus Enterotoxin A by ELISA

*S. aureus* fluid cultures were centrifuged for 10 min at 3500× *g* and 10 °C. Using a 0.85% NaCl solution, the supernatant was diluted to 1:100, 1:200 and 1:500 (*v*/*v*). Culture supernatants with SEA standard (0.1 ng/mL, Toxin Technology Inc., Sarasota, FL, USA) were plated (100 μL/well) at appropriate dilutions. Each well’s optical density (OD405) was measured using a microplate reader (Multiskan Ascent, Thermo Electron Corporation, Waltham, MA, USA). The OD_405_ readings were plotted versus the concentrations of SEA. Linear regression was used to calculate SEA concentrations. To ensure the reliability of results, three replicate measurements were acquired. The test was carried out according to the manufacturer’s recommendations [32].

### 2.8. Determination of the Effects of ZnO NPs against S. aureus Enterotoxin A (sea) Gene by PCR

The *S. aureus* enterotoxin A (*sea*) gene was identified utilizing specific primers, as shown in Table 2. DNA extraction was carried out based on Valihrach et al. [21], and the amplification reaction was carried out according to Rall et al. [33].

### 2.9. The Effects of ZnO NP on Bacterial Cell Morphology

The morphological characteristics of bacterial cells before and after treatment with ZnO NPs of size 20 nm (10 mM) were examined using a scanning electron microscope (SEM, S-500, Hitachi, Tokyo, Japan) [34]. Cells were primarily fixed with a 2.5% glutaraldehyde, 2% paraformaldehyde in 0.1 M Na-Cacodylate buffer, pH 7.35, for 30 min. The samples were then rinsed with ultra-pure water, dehydrated with a series of ethanol solutions, mounted onto SEM stubs, sputter-coated with gold/palladium and then examined.

### 2.10. Statistical Analysis

The obtained results were evaluated and interpreted using the Analysis of Variance (ANOVA) test, according to Feldman et al. [35].

## 3. Results

### 3.1. PCR Detection of nuc Gene

**Figure 1:**
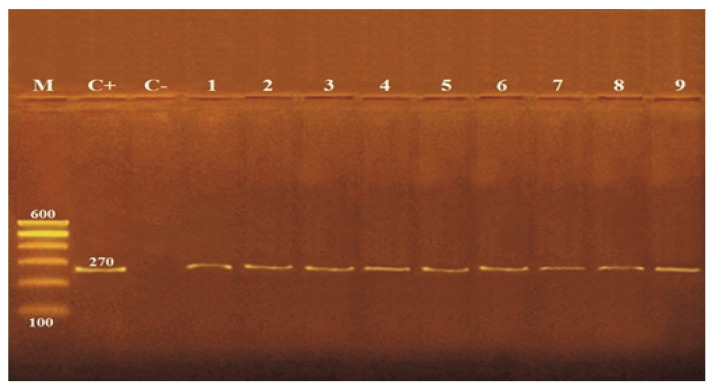
Detection of *nuc* gene (270 bp) by agarose gel electrophoresis of PCR amplified product. M: molecular wight marker, C+: control positive *S. aureus* for *nuc* gene., C−: control negative *S. aureus*, 1: untreated *S. aureus*, 2: 5 mM ZnO NPs (50 nm) with 10^5^ CFU/mL *S. aureus*, 3: 5 mM ZnO NPs (20 nm) with 10^5^ CFU/mL *S. aureus*, 4: 5 mM ZnO NPs (50 nm) with 10^7^ CFU/mL *S. aureus*, 5: 5 mM ZnO NPs (20 nm) with 10^7^ CFU/mL *S. aureus*, 6: 10 mM ZnO NPs (50 nm) with 10^5^ CFU/mL *S. aureus*, 7: 10 mM ZnO NPs (20 nm) with 10^5^ CFU/mL *S. aureus*, 8: 10 mM ZnO NPs (50 nm) with 10^7^ CFU/mL *S. aureus*, 9: 10 mM ZnO NPs (20 nm) with 10^7^ CFU/mL *S. aureus*.

PCR amplification of the *nuc* gene was confirmed by detecting 270 bp fragments (Figure 1). The results showed that there was no effect on *nuc* gene for (10^5^, 10^7^ CFU/mL) *S. aureus* treated with ZnO NPs at various sizes (20 and 50 nm) and concentrations (5 and 10 mM).

### 3.2. Antimicrobial Activity of ZnO NPs

The antimicrobial action on ZnO NPs on *S. aureus* showed that with the rise of ZnO NPs concentration and decrease of the size of the nanoparticles, the zone of inhibition increases, as demonstrated in Table 3.

### 3.3. Minimum Inhibitory Concentration (MIC)

The MIC of ZnO NPs at different sizes (20 and 50 nm) against 10^5^ CFU/mL *S. aureus* were 2.5 and 5 mM, respectively. While MIC with 10^7^ CFU/mL *S. aureus* was 5 mM for two sizes, as demonstrated in Table 4.

### 3.4. Growth Inhibitory Effects of ZnO NPs against S. aureus

Counts of *S. aureus* were determined before and after exposure to ZnO NPs at different sizes and concentrations. It was noticed that with increasing concentration and decreasing size of the nanoparticles, growth inhibition of *S. aureus* increased, resulting in the highest growth reduction of 98.99% with 10^5^ CFU/mL *S. aureus* against 20 nm and 10 Mm of ZnO NPs as shown in Table 5.

The correlation coefficient between the control vs. ZnO NPs treated cells concerning *S. aureus* counts resulted in the highest significant correlation for ZnO NPs of smaller size and higher concentration with the lowest initial cell concentration leading to lowering average growth rate compared to unexposed bacteria as shown in Table 6.

### 3.5. The effects of ZnO NPs against S. aureus Enterotoxin A by ELISA

The ELISA revealed a significant decrease in the concentrations of detectable enterotoxin A with all sizes (20 and 50 nm) and concentrations (5 and 10 mM) of ZnO NPs tested against 10^5^ and 10^7^ CFU/mL *S. aureus*. The reduction percent in concentrations of *S. aureus* enterotoxin A increased with a smaller size (20 nm) and higher concentration (10 mM) of ZnO NPs with rapid enterotoxin A clearance with a reduction percent reaching 100% as illustrated in Table 7.

The correlation coefficient between the untreated vs. ZnO NPs treated cells concerning *S. aureus* enterotoxin A concentrations resulted in a highly significant correlation due to a marked reduction in the concentrations of obvious enterotoxin A with ZnO NPs of smaller size and higher concentration with the lowest initial cell concentration compared to those of the nanoparticle-free control as shown in Table 8.

Particle size and concentration of ZnO NPs directly influenced counts and enterotoxin A concentrations of *S. aureus* as smaller size and higher concentration of ZnO NPs resulted in a significant reduction in measured amounts of enterotoxin A and counts of tested bacteria compared to untreated bacteria as shown in Table 9.

### 3.6. Antibacterial Activity of ZnO NPs against S. aureus Enterotoxin A (sea) Gene by PCR

**Figure 2:**
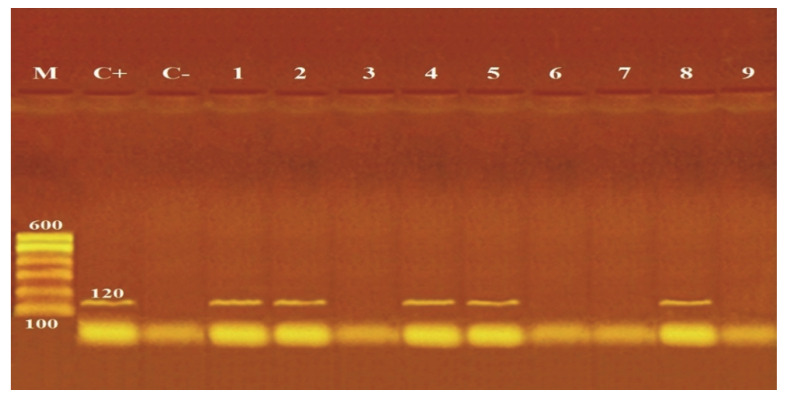
Detection of *sea* gene (120 bp) by agarose gel electrophoresis of PCR of the amplified product. M: molecular wight marker, C+: control positive *S. aureus* for *sea* gene, C−: control negative *S. aureus*, 1: untreated *S. aureus*, 2: 5 mM ZnO NPs (50 nm) with 10^5^ CFU/mL *S. aureus*, 3: 5 mM ZnO NPs (20 nm) with 10^5^ CFU/mL *S. aureus*, 4: 5 mM ZnO NPs (50 nm) with 10^7^ CFU/mL *S. aureus*, 5: 5 mM ZnO NPs (20 nm) with 10^7^ CFU/mL *S. aureus*, 6: 10 mM ZnO NPs (50 nm) with 10^5^ CFU/mL *S. aureus*, 7: 10 mM ZnO NPs (20 nm) with 10^5^ CFU/mL *S. aureus*, 8: 10 mM ZnO NPs (50 nm) with 10^7^ CFU/mL *S. aureus*, 9: 10 mM ZnO NPs (20 nm) with 10^7^ CFU/mL *S. aureus*.

There was a marked effect on *sea* gene in reaction to ZnO NPs with various sizes (20 and 50 nm) and concentrations (5 and 10 mM), as shown in Figure 2. Gene absence could be detected for 10^5^ CFU/mL *S. aureus* treated with 20 nm (5 and 10 mM) and 50 nm (10 mM) ZnO NPs, also with 10^7^ CFU/mL *S. aureus* treated with 20 nm (10 mM) ZnO NPs. While with other sizes and concentrations of ZnO NPs, the presence of *sea* gene was not affected.

### 3.7. Effects of ZnO NPs on S. aureus Morphology by SEM

**Figure 3:**
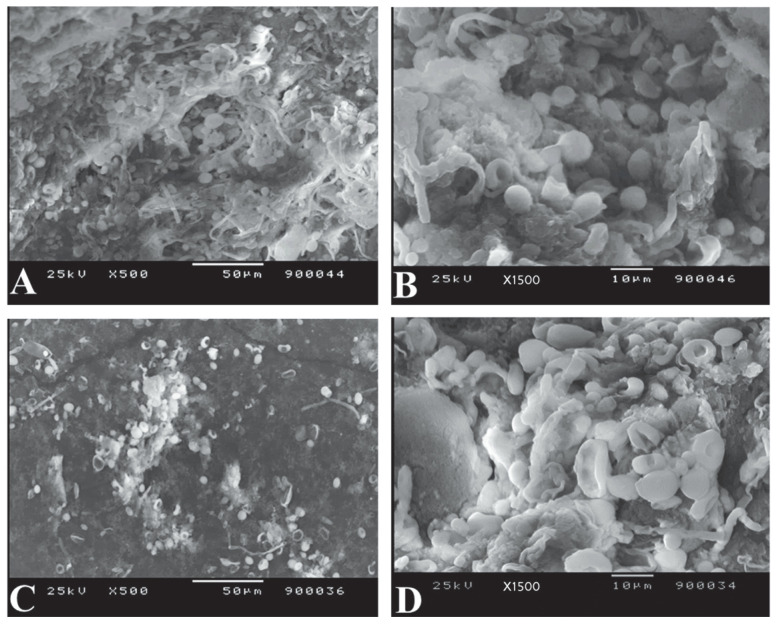
Scanning Electron Microscopic examination of bacterial morphology of *S. aureus* (**A**,**B**): without ZnO NPs treatment exhibiting a spherical, smooth appearance and cells that were nearly identical in shape (**C**,**D**): with ZnO NPs treatment showing abrupt morphology with damaged cell surface.

*S. aureus* normally exhibited a spherical, smooth morphology, with cells that were nearly identical in shape (Figure 3A,B). After exposure to ZnO NPs of size 20 nm (10 mM), the cells exhibited abrupt morphological and surface damage (Figure 3C,D).

## 4. Discussion

*S. aureus* is a significant pathogen that can cause a wide range of infections in humans and animals, such as superficial and systemic infections [18]. Metal-based nanoparticles have been intensively studied for several biomedical uses. Based on the World Health Organization, metal-based nanoparticles were proven to be efficient against pathogens listed as a priority, additionally to their decreased size and choosiness for bacteria [13]. Since the antibiotic resistance in *S. aureus* is rapidly increasing, ZnO NPs can be considered as a treatment [36].

In this study, an agar gel diffusion test assessed ZnO NPs in vitro for their antibacterial action on S. aureus. Comparison between sizes (20 and 50 nm) with various concentrations (2.5, 5, 10 and 20 mM), ZnO NPs size and concentration highly influenced the bacterial growth. It resulted in 26 and 22 mm inhibitory zones at a smaller size (20 nm) at high concentrations (20 mM) of ZnO NPs against 10^5^ and 10^7^ CFU/mL *S. aureus*, respectively. These findings are quite similar to those detected by Gunalan et al. [37], who reported that nano and bulk ZnO NPs showed antibacterial action on *S. aureus* and maximum activity 26/23 mm was detected for size 25 nm and concentrations of 2, 4 and 6 mM ZnO NPs. Moreover, Mirhosseini and Firouzabadi [38] reported that the inhibition zone of ZnO NPs size (20–25 nm) and concentrations of 2, 5 and 10 mM against 10^7^ CFU/mL *S. aureus* showed no inhibition zone, 10 mm and 12 mm, respectively. While our results showed 0, 8 and 16 mm inhibition zones for ZnO NPs of size 20 nm against 10^7^ CFU/mL *S. aureus*. The results proved that smaller size ZnO NPs and higher concentrations showed more effective antimicrobial growth inhibition.

The findings in Table 4 revealed that MIC of the tested ZnO NPs (20, 50) nm against 10^5^ CFU/mL *S. aureus* were 2.5 and 5 mM, respectively. While against 10^7^ CFU/mL *S. aureus* was 5 mM for both sizes. This disagreed with prior published studies on the antibacterial characteristics of ZnO NPs as Gunalan et al. [37] reported 0.8 mM as MIC value of ZnO NPs with size 25 nm against *S. aureus*. Tayel et al. [28] showed that ZnO NPs with average size of 50 nm had MIC values against *S. aureus* of 10 mM, which is higher compared to the values found in the current study. Moreover, Abd EL-Tawab et al. [39] reported an 18.4 mM MIC of 19 nm ZnO NPs that inhibited the growth of S. aureus, per the current investigation. The collective MIC results were different from those obtained in the present study suggesting that the antimicrobial action of ZnO NPs may be affected by the preparing technique, particle size and concentration. Furthermore, the difference in MIC results against the test microorganisms might be due to the differences in strains used and/or test conditions.

It was obvious that the mean values of *S. aureus* markedly reduced after treatment with 20 nm ZnO NPs at 5 and 10 mM with 10^5^ CFU/mL *S. aureus* with high growth reduction of 89.72%, and 98.99%, respectively. While with 10^7^ CFU/mL *S. aureus*, growth reduction was 74.06% and 87.93% after treatment with 5 and 10 mM, respectively. Furthermore, the 50 nm ZnO NPs at 5 and 10 mM showed lower growth reduction of 80.97% and 86.67%, respectively, with 10^5^ CFU/mL *S. aureus*. But with 10^7^ CFU/mL *S. aureus*, the growth reduction decreased to 62.51% and 69.88%, respectively (Table 5). Our data demonstrated that the highest inhibitory action of ZnO NPs was achieved by smaller size 20 nm with a higher concentration 10 mM against 10^5^ CFU/mL *S. aureus*. The results were concordant with Saafan et al. [40], who reported that size 20 nm achieved a high growth reduction 97.49% and 99.10% for *S. aureus* with 5 and 10 mM ZnO NPs, respectively at 24 h. However, decreased outcomes were achieved by Ibrahim et al. [41], who reported that the highest growth reduction for *S. aureus* (10^7^ CFU/mL) treated with a concentration 10 mM at sizes 50 and 20 nm ZnO NPs was 12.56% and 25.35%, respectively. The results here with Mirhosseini and Firouzabadi [38] showed that 20–25 nm at 5 mM ZnO NPs had a 33.9% growth decrease, which is lower than our results, while showing agreement with significant growth inhibition by 10 mM, which nearly totally stopped the growth of 10^7^ CFU/mL *S. aureus* at 24 h. The probable mechanisms of action of metal nanoparticles could be: (a) excessive generation of reactive oxygen species within bacteria, (b) disturbance of key enzymes in the respiratory chain through microbial plasma membranes damage, (c) metal ion collecting in microbial membranes, (d) electrostatic attraction among nanoparticles of metal and microbial cells inhibits metabolic processes, and (e) suppression of microbial proteins/enzymes through improved production of H2O2 [42].

These findings imply that bacterial growth depends entirely on ZnO NPs concentrations and an initial number of bacterial cells. As a result, the antibacterial action of ZnO NPs could be size-dependent.

The correlation coefficient between the control vs. ZnO NPs treated cells concerning S. aureus counts resulted in the highest significant correlation (+0.76) for ZnO NPs of smaller size (20 nm) and higher concentration (10 mM) with the lowest initial cell concentration (10^5^ CFU/mL) leading to lowering average growth rate compared to unexposed bacteria as shown in Table 6.

Moreover, the results showed that sea produced at varying levels control with high production capacity compared with *S. aureus* treated with ZnO NPs. The mean value of the produced amount of sea was 0.93 ± 0.18 ng/mL for 10^5^ CFU/mL *S. aureus* treated with ZnO NPs 50 nm (5 mM) with a reduction percent (75.71%). On the contrary, ZnO NPs at 50 nm (10 Mm) and 20 nm (5 and 10 mM) achieved 100% reduction percent. They inhabited sea production, while the mean value of the high produced amount of sea was 4.01 ± 0.56 ng/mL for 10^7^ CFU/mL *S. aureus* and treated with ZnO NPs 50 nm (5 mM) with the lowest reduction percent (58.74%), unlike size 20 nm (10 mM) that completely prevented sea production with 100% reduction percent as shown in Table 7. These results demonstrate that nanoparticles are size and concentration-dependent and can effectively inhibit or clear enterotoxin. Similarly, Li et al. [43] employed a commercial enterotoxin ELISA kit to determine enterotoxin concentration with or without the treatment of protease-conjugated gold nanorods (PGs). Due to protease breakdown by bacterial extracellular enzymes, protease alone had only a moderate inhibitory effect on enterotoxins release. They also looked at the ability of PGs to remove existing enterotoxins. The results demonstrated that the effects of PGs were dose-dependent and produced fast enterotoxin clearance, with 86.5% of enterotoxin content removed after 10 minutes of near-infrared (NIR) laser illumination and treatment with 200 µg/mL PGs. Moreover, the results here agreed with those found by Findlay et al. [44] that employed ELISA to identify measurable concentrations of LL-37 (human cathelicidin) after exposure to carbon black nanoparticles. All carbon black nanoparticle concentrations studied (25, 50 and 100 mg/mL) resulted in a substantial drop in detectable LL-37 concentrations, with samples containing both LL-37 and carbon nanoparticles detected at similar protein concentrations to those of the nanoparticle-only controls.

The correlation coefficient between the untreated vs. ZnO NPs treated cells concerning *S. aureus* enterotoxin A concentrations resulted in a highly significant correlation (+ 0.81) due to a marked decrease in the concentrations of detectable enterotoxin A with ZnO NPs of smaller size (20 nm) and higher concentration (10 mM) with lowest initial cell concentration (10^5^ CFU/mL) compared to those of the nanoparticle-free control as shown in Table 8.

Particle size and concentration of ZnO NPs directly influenced counts and enterotoxin A concentrations of *S. aureus* as smaller size (20 nm) and higher concentration (10 mM) of ZnO NPs resulted in a significant reduction in measured amounts of enterotoxin A and counts of tested bacteria with lowest initial cell concentration (10^5^ CFU/mL) compared to untreated bacteria with high significant correlation (+ 0.89) as shown in Table 9.

PCR for the *nuc* gene confirmed the presence of *S. aureus* DNA in control and treated cells (10^5^, 10^7^ CFU/mL) with different sizes (20 and 50 nm) and concentrations (5 and 10 mM) of ZnO NPs, revealing a lack of effect on *nuc* gene (Figure 1). On the contrary, PCR detection of the *sea* gene in control and ZnO NPs treated cells with different sizes (20 and 50 nm) and concentrations (5 and 10 mM) showed variable effects of *sea* gene in response to ZnO NPs exposure. Loss of *sea* gene was detected with 10^5^ CFU/mL *S. aureus* treated with 20 nm (5 and 10 mM) and 50 nm (10 mM) ZnO NPs and 10^7^ CFU/mL *S. aureus* treated with 20 nm (10 mM) ZnO NPs (Figure 2). This explains the reduction of *sea* production caused by ZnO NPs treatments (Table 7). The findings of our investigation indicated that ZnO NPs could inhibit *sea*, leading to a decrease or loss of pathogenic properties. This may be due to the higher concentration and smaller size of ZnO NPs, which can function as a genotoxic agent and cause DNA damage at priming sites, inhibiting virulence factors’ expression. In accordance, Salama et al. [45] reported that the genotoxic effect of ZnO NPs on the genomic DNA of common dry bean significantly impacts the expression of genes encoding specific proteins. Applying ZnO NPs may trigger the inactivation of certain genes, resulting in the absence of specific proteins. Moreover, Ghosh et al. [46] noted that at high concentrations of ZnO NPs, the root meristems of Allium cepa cells lost their membrane integrity, chromosome abnormalities increased, and DNA strands broke.

Furthermore, Saghalli et al. [47] demonstrated that ZnO NPs at sub-MIC dosages could decrease *S. aureus* hemolysis gene expression. However, this effect was not observed when the nanoparticle dose was less than 1/4 of the MIC. Their results revealed that expression of the hemolysin gene during the stationary phase was about 4.8 times lower than in the log phase, explaining the termination of hemolysis in a 24 h culture. In another study, Moghassem-Hamidi et al. [48] concluded that there was no link between the existence of the thermonuclease gene and the generation of enterotoxin.

The impacts of ZnO NPs on the cellular structure of *S. aureus* by SEM (Figure 3) demonstrated that incubating bacterial cells with ZnO NPs of size 20 nm (10 mM) causes morphological alterations and cell shape distortion owing to cell membrane disruption. Meanwhile, the control sample retained a round, smooth morphology of almost similar size and no surface damage. The results obtained by Abd EL-Tawab et al. [39], Manzoor et al. [49], Rauf et al. [50], Rauf et al. [51] and Mohd Yusof et al. [52] confirmed the morphological changes of *S. aureus* after treatment with ZnO NPs. This could also explain the reduction in growth rate and loss of *sea* gene of *S. aureus* due to cell destruction by ZnO NPs treatments.

## 5. Conclusions

In conclusion, the ZnO NPs possess significant antibacterial properties. ZnO NPs of 20 nm particle size suppressed the growth of enterotoxigenic *S. aureus* better, and their inhibitory effects improved when ZnO NPs concentration was increased. The ZnO NPs had greater efficacy in inhibiting enterotoxin A production of *S. aureus* where the *sea* gene was significantly affected. Furthermore, these findings indicate that ZnO NPs could be a potential anti-virulence agent against enterotoxigenic *S. aureus.*

## Figures and Tables

**Table 1:** Oligonucleotide primers sequences used to detect *nuc* gene.

Target Gene	Oligonucleotide Sequence (5′→3′)	Product Size (bp)	References
*nuc* (F)	5′ GCGATTGATGGTGATACGGTT 3′	270	Brakstad et al. [23]
*nuc* (R)	5′ AGCCAAGCCTTGACGAACTAAAGC 3′		

**Table 2:** Oligonucleotide primers sequences used for detection of *sea* gene.

Target Gene	Oligonucleotide Sequence (5′→3′)	Product Size (bp)	References
*sea* (F)	5′ TTGGAAACGGTTAAAACGAA 3′	120	Rall et al. [33]
*sea* (R)	5′ GAACCTTCCCATCAAAAACA 3′		

Oligonucleotide Sequence: http://www.ncbi.nlm.nih.gov/pmc/articles/PMC140333/table/t2/; accessed on 1 October 2022.

**Table 3:** Antimicrobial activity of ZnO NPs at different sizes and concentrations on *S. aureus*.

*S. aureus*	Different Sizes of ZnO NPs (nm)	Diameter of the Inhibition Zone (mm) in Different Concentrations of ZnO NPs			
		20	10	5	2.5
10^5^ CFU/mL	Control	0	0	0	0
	50	18	12	8	0
	20	26	22	16	6
10^7^ CFU/mL	Control	0	0	0	0
	50	16	6	5	0
	20	22	16	8	0

**Table 4:** MIC of the tested ZnO NPs on *S. aureus*.

*S. aureus*	Different Sizes of ZnO NPs (nm)	MIC (mM)
10^5^ CFU/mL	Control	0
	50	5
	20	2.5
10^7^ CFU/mL	Control	0
	50	5
	20	5

**Table 5:** Growth inhibition of *S. aureus* by ZnO NPs.

*S. aureus*	ZnO NPs Conc. (mM) ZnO NPs Size (nm)	5		10	
		Count	R% *	Count	R%
10^5^ CFU/mL	Control	9.93 × 10^4^ ± 0.81 × 10^4^	0	9.83 × 10^4^ ± 0.92 × 10^4^	0
	50	1.89 × 10^4^ ± 0.25 × 10^4^	80.97	1.31 × 10^4^ ± 0.14 × 10^4^	86.67
	20	1.02 × 10^4^ ± 0.09 × 10^4^	89.72	9.97 × 10^2^ ± 0.86 × 10^2^	98.99
10^7^ CFU/mL	Control	9.87 × 10^6^ ± 0.95 × 10^6^	0	9.86 × 10^6^ ± 0.79 × 10^6^	0
	50	3.70 × 10^6^± 0.44 × 10^6^	62.51	2.97 × 10^6^ ± 0.36 × 10^6^	69.88
	20	2.56 × 10^6^ ± 0.27 × 10^6^	74.06	1.19 × 10^6^ ± 0.18 × 10^6^	87.93

* R% = Reduction%.

**Table 6:** Correlation coefficient between the control vs. ZnO NPs treated cells with respect to *S. aureus* counts.

ZnO NPs Conc.(mM) ZnO NPs Size (nm)	5		10	
	*S. aureus* 10^5^ CFU/mL	*S. aureus* 10^7^ CFU/mL	*S. aureus* 10^5^ CFU/mL	*S. aureus* 10^7^ CFU/mL
50	+0.49	+0.37	+0.58	+0.43
20	+0.65	+0.51	+0.76	+0.62

**Table 7:** Reduction of *S. aureus* enterotoxin A concentrations by ZnO NPs treatments.

*S. aureus*	ZnO NPs Conc.(mM) ZnO NPs Size(nm)	5		10	
		SEA Concentration	R% *	SEA Concentration	R%
10^5^ CFU/mL	Control	3.83 ± 0.42	0	3.37 ± 0.29	0
	50	0.93 ± 0.18	75.71	0	100
	20	0	100	0	100
10^7^ CFU/mL	Control	9.72 ± 0.80	0	9.05 ± 0.73	0
	50	4.01 ± 0.56	58.74	1.67 ± 0.24	81.56
	20	0.54 ± 0.07	94.44	0	100

* R% = Reduction%.

**Table 8:** Correlation coefficient between the untreated vs. ZnO NPs treated cells with respect to *S. aureus* enterotoxin A concentrations.

ZnO NPs Conc.(mM) ZnO NPs Size(nm)	5		10	
	*S. aureus* 10^5^ CFU/mL	*S. aureus* 10^7^ CFU/mL	*S. aureus* 10^5^ CFU/mL	*S. aureus* 10^7^ CFU/mL
50	+0.56	+0.43	+0.69	+0.50
20	+0.74	+0.60	+0.81	+0.71

**Table 9:** Correlation coefficient between *S. aureus* counts vs. enterotoxin A concentrations for untreated and ZnO NPs treated cells.

*S. aureus*	ZnO NPs Conc.(mM) ZnO NPs Size (nm)	5	10
10^5^ CFU/mL	Control	+0.61	+0.66
	50	+0.74	+0.80
	20	+0.82	+0.89
10^7^ CFU/mL	Control	+0.54	+0.57
	50	+0.69	+0.76
	20	+0.78	+0.85

## Data Availability

Not applicable.
